# Zukunft unter Klima-Unsicherheiten agil und nachhaltig gestalten

**DOI:** 10.1007/978-3-662-62195-0_11

**Published:** 2020-10-26

**Authors:** Sebastian Abel, Jakob Michelmann

**Affiliations:** Institut für Innovation und Technik, Berlin, Germany; Institut für Innovation und Technik, VDI/VDE Innovation + Technik GmbH, 10623 Berlin, Deutschland

## Abstract

Der beschleunigte Klimawandel fordert Wirtschaft und Gesellschaft heraus, zu handeln, um Klimaveränderungen abzuschwächen und zu kontrollieren sowie unser Leben an die sich ändernden Umstande anzupassen. Dabei trifft hoher Entscheidungsdruck auf große Unsicherheit. Um Handlungsmöglichkeiten zu erkennen und zu ergreifen, gilt es, die Instrumente für eine agile strategische Orientierung und Gestaltung im Klimawandelzeitalter zu sortieren und zu scharfen.

Der Klimawandel und die Klimapolitik sind Treiber eines neuen Strukturwandels mit sehr unterschiedlichen potenziell negativen wie positiven wirtschaftlichen und gesellschaftlichen Auswirkungen (vgl. Bardt et al. 2012:29). Im Zentrum steht die wissenschaftliche Erkenntnis, dass selbst im Falle des sogenannten „Best-Case-Szenarios" – also einer Limitierung des globalen Temperaturanstiegs auf 1,5 bis 2 Grad Celsius – mit sehr einschneidenden Veränderungen unserer Lebensbedingungen zu rechnen sein wird. So wird es künftig ein „stabiles" Klima als Planungsgrundlage etwa in der Landwirtschaft nicht mehr im bekannten Maße geben. Auch ist von Kaskadeneffekten in unseren Ökosystemen auszugehen (vgl. IPCC 2019:15), die aufgrund ihrer Komplexität und zahlreich stattfindender Wechselwirkungen kaum vorhersehbar sind.

Kurzum: Der Klimawandel und die gesellschaftliche Reaktion darauf werden keinen Bereich in Wirtschaft und Gesellschaft unberührt lassen und vieles fundamental verändern. Aber was tun? Entscheidungsträger:innen in Unternehmen, Politik und Verwaltung verspüren große Unsicherheit. Um mögliche Entwicklungen besser zu verstehen und erfolgreiche Strategien zur Bewältigung des Klimawandels zu entwickeln, ist eine Denk- und Handlungsweise vonnöten, mit der potenzielle zukünftige Zusammenhänge aufgespürt und alternative Handlungsoptionen erkannt und genutzt werden können, anstatt „die" Zukunft als gegeben hinzunehmen (vgl. Slaughter 2002:3). Es geht darum, die Zukunft nachhaltig auf die Probe zu stellen: Mit einer agilen strategischen Vorausschau zur Orientierung und zur Gestaltung des Klimawandelzeitalters.

Die Art und Weise und die Komplexität der Betroffenheit wird stark variieren. In Mitteleuropa ist eher von einer indirekten Betroffenheit durch den Klimawandel auszugehen, wie Abb. 11.1 für die Wirtschaft illustriert.

| | Ausprägung | Beispiel |
|---|---|---|
| **Direkte Betroffenheit** | Natürlich-physikalische Betroffenheit | • Starkregen oder Dürre beeinflussen landwirtschaftliche Produktion<br>• Extremwetterphänomene beschädigen Infrastruktur<br>• Hohe Temperaturen erhöhen den Energiebedarf |
| **Indirekte Betroffenheit** | Regulatorische Betroffenheit | • Regulierung energieintensiver Unternehmen (z. B. Zement-, Metall- oder Papierherstellung) sowie der Energieindustrie (bspw. $CO_2$-Bepreisung)<br>• Veränderte Vorschriften der Bauleitplanung |
| | Marktliche Betroffenheit (Beschaffung) | • Zulieferbetriebe sind direkt oder indirekt betroffen und können (Vor-)Produkte oder Rohstoffe nicht, eingeschränkt oder zu steigenden Preisen liefern |
| | Marktliche Betroffenheit (Absatz) | • Produktabnehmer:innen sind direkt oder indirekt betroffen und fallen als Kund:innen (teilweise) aus (z. B. in der Automobilindustrie)<br>• Kaufverhalten von Konsument:innen verändert sich, „klimafreundliche" Produkte werden präferiert |

*Abb. 11.1 Kategorisierung der verschiedenen Formen der Betroffenheit von Unternehmen durch den Klimawandel angelehnt an Mahammadzadeh (2012)*

Wie Bardt et al. (2012:30 ff.) verdeutlichen, sind auch Unternehmen von klimapolitisch bedingten Risiken in unterschiedlicher Weise betroffen. So kann eine starke Außenhandelsintensität gerade für deutsche Unternehmen ein Risikofaktor sein, wenn etwa Regulierungen hierzulande Produktionskostensteigerungen zur Folge haben, die im internationalen Wettbewerb nicht oder nur selten an Kunden weitergegeben werden können. Auch können sich internationale Lieferketten aufgrund direkter Betroffenheit als wenig klimaresilient erweisen. Allerdings tun sich in der Wirtschaft auch Chancen auf: Beispielsweise können „klimaeffiziente" Produkte und Innovationen Unternehmen zu einer stärkeren Wettbewerbsposition verhelfen oder öffentlich geförderte Investitionsanreize nachhaltige Modernisierungen ermöglichen.

Auch in den Kommunen wächst der Handlungsdruck (s. Kap. 5: Klimafreundliche Kommunen). Zwar betreiben viele von ihnen schon seit längerer Zeit aktiven Klimaschutz, indem sie etwa in erneuerbare Energien investieren. Zugleich müssen sie sich jedoch auf immer gravierendere Umfeldveränderungen und Klimaschutzanforderungen einstellen. Direkte Betroffenheit, zum Beispiel aufgrund zunehmender Hitze in Städten, Stürme oder Starkregen, trifft auf indirekte Betroffenheit, beispielsweise durch neue Anforderungen an die Gesundheitsversorgung oder wirtschaftliche Effekte wie sich verändernde Steuereinnahmen. Eine besondere Herausforderung für kleinere und mittlere Kommunen ist die Bereitstellung von hier naturgemäß knappen Ressourcen und Kapazitäten für den Klimaschutz in Konkurrenz zu den alltäglichen Aufgaben (vgl. Schüle et al. 2016:10 ff.).

Angesichts der zahlreichen und komplexen Veränderungsimpulse wie politischen Maßnahmen (z. B. $CO_2$-Bepreisung oder Investitionsanreize für klimafreundliche Technologien), technologischen Innovationen (z. B. im Bereich der Digitalisierung) oder sozialen Entwicklungen (z. B. ein verändertes Konsumentenverhalten, soziale Bewegungen) sehen sich viele Akteur:innen in Wirtschaft und Verwaltung überfordert. Kausalitäten können nicht in Gänze nachvollzogen und die Konsequenzen von Handlungen, die vielleicht erst Jahre später sichtbar werden, nicht im Vorfeld abgeschätzt werden (vgl. Beck 2007:63 ff.). Im Nachhinein sind Zusammenhänge oft leicht erkennbar, jedoch im Vorfeld meist nur von Expert:innen als schwache Signale zu identifizieren (vgl. Steinmüller und Steinmüller 2004:23 ff.). Diese Handlungsunsicherheiten fußen auch auf einer wahrgenommenen Unsicherheit, da sich unser Bewusstsein für Risiken durch die Wissenschaft und mediale Kommunikation erhöht hat (vgl. Beck 2007:65 f.)

## *Strategische Vorausschau*

Viele Akteur:innen sehen die Strategieentwicklung als ein Mittel, um diese Unsicherheit zu überwinden. Dies soll gelingen, indem sie feste Meilensteine definieren. Anhand dieser werden Maßnahmen und Handlungen definiert. Die Strategieentwicklung ist in größeren Unternehmen und Organisationen vorwiegend in eigenen Einheiten institutionalisiert, wobei deren Prozesse häufig stark formalisiert ablaufen. Die so entwickelten Strategien sind oft langfristig ausgerichtet und gehen von einer eher „linearen" Zukunft aus. Alternative oder wenig wahrscheinliche zukünftige Entwicklungen werden nur selten betrachtet – mit der Folge, dass Strategien häufig unterkomplex und unflexibel sind. Darüber hinaus ist Strategiearbeit meist von einer empirisch quantifizierbaren, technologisierten Weltanschauung geprägt und auf Schlüsselindikatoren wie Wachstumsraten oder Ressourceneffizienz ausgerichtet (vgl. Slaughter 2002:1). Unternehmen und Kommunen, die an einmal gesetzten Meilensteinen trotz sich ändernder Umfeldentwicklungen festhalten, können in diesen hoch dynamischen Zeiten riskieren, wichtige Anpassungsschritte zu versäumen.

Methoden der strategischen Vorausschau – wie bereits von einigen Organisationen im strategischen Management angewendet – können dabei helfen, derart lineare Fokussierungen zu einer integrativen Perspektive zu erweitern, die eine größere Offenheit zukünftiger Entwicklungen antizipiert und dadurch die Nachhaltigkeit des eigenen Handelns fördert (vgl. Slaughter. 2002:1). Die Vielzahl der denkbaren äußeren

Entwicklungen, aber auch der eigenen Gestaltungsmöglichkeiten, macht deutlich, dass strategische Vorausschau nicht „die" Zukunft zum Gegenstand ihrer Untersuchungen erheben kann – sondern mögliche, wünschenswerte oder wahrscheinliche „Zukünfte" (vgl. Schüll 2009:14, Herv. d. V.; vgl. Kreibich 2006:3).[70] Strategische Vorausschau erarbeitet Orientierungswissen für die Strategieentwicklung, um sich auf eine Vielzahl unterschiedlicher Zukünfte vorzubereiten und damit zukunftsfest aufzustellen. Strategische Vorausschau kann einerseits als Prozess verstanden werden, in dem fundierte Zukunftsbilder[71] identifiziert werden, um Entscheidungsoptionen abzuleiten (vgl. Voros 2005:10 f.). Sie kann anderseits auch als erlernbare Fähigkeit einer vorwärtsgerichteten Denkweise angesehen werden: das Denken in Alternativen (vgl. Slaughter 2002:1).

Als zentrale Hemmnisse für die Entwicklung von Anpassungsstrategien erweisen sich neben Wissensdefiziten vor allem nicht ausreichende Anpassungskapazitäten wie fehlende finanzielle Mittel (vgl. Bardt et al. 2012:35). Auch bedarf es unterschiedlicher Fähigkeiten innerhalb einer Organisation, um adäquat auf komplexe Herausforderungen reagieren zu können, insbesondere die Beteiligung der Bereiche Risikoabschätzung, Kommunikation nach außen und nach innen sowie Management und Strategie (vgl. Okereke et al. 2012:13).

Zur Fähigkeit, Anpassungsstrategien zu entwickeln, zählt auch die Kompetenz, Pfadabhängigkeiten der eigenen Organisation zu erkennen. Pfade sind im Sinne technischer und organisatorischer Pfadtheorien retrospektiv nachvollziehbare Entwicklungen entlang eines Entwicklungsmusters (vgl. Arthur 1989:122; vgl. David 1998:4; vgl. Dievernich 2012:64). Ein Beispiel hierfür ist der Verbrennungsmotor, der seit mehr als einhundert Jahren seinen Einsatz in Fahrzeugen findet. Pfade können jedoch auch Entscheidungsmuster sein, wie die Managemententscheidung eines ÖPNV-Betreibers, weiterhin vor allem in Fahrzeugmodelle mit Verbrennungs-

[70] *So mag es wahrscheinlich sein, dass bis 2030 in Europa der Fahrzeugbestand insgesamt und mit überwiegendem Anteil an fossilen Energieträgern steigt (Statista 2020; ERTRAC 2017: 37) (wahrscheinliche Zukunft). Um Klimaziele zu erreichen, wäre ein rapider Flottenwandel hin zu alternativen Antrieben mit erneuerbaren Energien bei gleichzeitiger Verringerung der Flottengröße in Europa wünschenswert (wünschenswerte Zukunft). Dass sich das Mobilitätsverhalten vieler Menschen schlagartig, wie krisenbedingt durch COVID-19, in den virtuellen Raum verlagern kann, deutet daraufhin, dass ähnliche soziale Entwicklungen auch im Zusammenhang mit klimabedingten Krisen möglich sind (mögliche Zukunft).*

[71] *Zukunftsbilder repräsentieren Zukünfte im Sinne der Offenheit der Zukunft (vgl. Grunwald 2009: 26), die noch nicht real, nicht empirisch überprüfbar und „auch anders möglich" sind (Neuhaus und Steinmüller 2015: 18).*

motor zu investieren statt in Strom- oder Wasserstoffantriebe. Erzwingen externe Faktoren wie Wettbewerbsdruck oder auch interne Faktoren wie vorangegangene Entscheidungen – sogenannte Lock-in-Mechanismen – das Fortsetzen eines Pfades, liegt eine Pfadabhängigkeit vor. Sie können „blind spots" hervorrufen, denn man kann „nicht sehen, dass man nicht sieht, was man nicht sieht" (Dievernich 2012:65). Das Verstehen der Umwelt und zukünftiger Entwicklungen allein reicht nicht aus, um Handlungsspielräume abzuleiten und Strategien zu entwickeln, denn Pfadabhängigkeiten engen den Blick auf alternative Entwicklungen der eigenen Organisation ein, während das Umfeld die Entwicklung anderer Pfade fordert. Organisatorische Lock-in-Mechanismen können zusammen mit äußeren Entwicklungen strategische Entscheidungen massiv beeinflussen. Gleichzeitig ist klar, dass die Handelnden nicht als Spielball in diesen deterministischen Strukturen gefangen sind, sondern ihre zukünftige Entwicklung im Rahmen ihres Handlungsspielraums selbst neu definieren und gestalten können. Diese Denk- und Handlungsweise ist Grundlage für die Anwendung von Methoden der strategischen Vorausschau (Dievernich 2012:67 f.).

Insbesondere größere Unternehmen und Konzerne haben entsprechende Instrumente teilweise schon in ihre Strategieprozesse integriert. Dabei nutzen sie verstärkt formalisierte und langfristig ausgerichtete Methoden wie Horizon Scanning, Delphi-Befragungen oder komplexe Szenarioanalysen (vgl. Slaughter 2002:2). Derartige zeitintensive Methoden können die Entscheidungsfindung allerdings nicht immer zeitnah und situativ unterstützen, was jedoch in einem sehr dynamischen Kontext, in dem kurzfristige Veränderungen wie technische Innovationen oder politische Entscheidungen große Auswirkungen haben können, gefordert sein kann. Vor diesem Hintergrund zeichnet sich das Dilemma ab, strategische Entscheidungen bei hoher Unsicherheit und Volatilität der Entwicklungen regelmäßig situationsbezogen anpassen zu müssen. Die Strategieentwicklung muss deshalb durch Methoden ergänzt werden, die es ermöglichen, auf derartige Veränderung agil zu reagieren (vgl. Prange 2018:3).

## *Agile strategische Vorausschau*

Der Begriff Agilität rekurriert auf agere (lat.): „machen", „tun". In der Debatte um agiles Management wird vorwiegend auf die Notwendigkeit für schnelles, flexibles Handeln in einem dynamischen Umfeld verwiesen. Allerdings ist das nur eine bestimmte Auslegung des Begriffs, der im Bereich der Softwareentwicklung geboren wurde. Schnelles, effektives Handeln bedarf nämlich auch des Reflektierens, dessen Ergebnis ebenso wie das Handeln das Abwarten sein kann. Andererseits sollen agile Methoden des Managements helfen, die Entscheidungsfähigkeit zu erhöhen (vgl. Prange 2018:8 f.). Agile Methoden der Vorausschau unterstützen beides gleichermaßen, das Reflektieren ebenso wie schnelle Entscheidungen und Reaktionen.

Generell können Methoden der strategischen Vorausschau nach unterschiedlichen Kriterien differenziert werden. Zum Beispiel ob sie explorativ potenzielle zukünftige Entwicklungen untersuchen oder ob sie etwa mögliche Handlungsoptionen im Hinblick auf normative, also gewünschte Ziele ergründen (vgl. Schüll 2009:224). Agile Methoden können sowohl explorativ als auch normativ ausgerichtet sein und lassen sich hinsichtlich Umfang und Aufwand abgrenzen:

- Thematischer Umfang: Dieser wird durch den gewünschten Grad des Themenhorizonts (eingeschränkt – offen) und die Detailtiefe (niedrig – tief) definiert und hängt vom Erkenntnisinteresse der Akteure und der Natur der zu untersuchenden Thematik ab (vgl. Hines und Bishop 2013:32 f.). Dabei bedeutet eine niedrigere Detailtiefe keinesfalls eine mindere Qualität. So gibt zum Beispiel das Erkenntnisinteresse vor, ob es wichtig ist, Projektionen auf Grundlage umfangreicher Berechnungen zu erstellen oder sie etwa aus einem strukturierten partizipativen Expertenaustausch plausibel abzuleiten.
- Aufwand und Zeitraum (Komplexität des Prozesses): Je nach Ausgestaltung des Vorausschauprozesses kann dieser kurzfristig oder aufgrund komplexer Abläufe, zum Beispiel bei der mehrstufigen Validierung der Resultate durch verschiedene Expertengremien, erst nach einem oder mehreren Jahren zu Ergebnissen führen. In diesem Zusammenhang spielt auch die sogenannte Formalität eine Rolle. Methoden werden in der Regel als systemisch-formal bezeichnet, wenn sie große, häufig quantitative Datenmengen maschinengestützt verarbeiten. Als eher informell-intuitiv gelten dagegen Methoden, die sich vor allem durch einen konstruierenden Charakter auszeichnen und sich besonders dazu eignen, qualitative, wissenschaftlich gestützte Aussagen zu zukünftigen Entwicklung zu machen (vgl. Kosow und Gaßner 2008:51; 69 f.). Der Grad der Formalität impliziert keine Wertung der wissenschaftlichen Qualität.

Das wichtigste Kriterium für die Entscheidung, klassische oder agile Methoden zu nutzen, ist der Zeitpunkt des angestrebten Ergebnisgewinns: Werden die Ergebnisse kurzfristig benötigt, um zum Beispiel auf ein gerade verabschiedetes Klimagesetz zu reagieren, oder liegt ein eher langfristiges Erkenntnisinteresse vor, um etwa sich langsam abzeichnende Trends über einen längeren Zeitraum zu ergründen? Da der thematische Umfang die Komplexität des Vorausschauprozesses vorgibt, ist es für agile Methoden entscheidend, dass sie eine eher geringe Detailtiefe anstreben und den Themenbezug beschränken.

Agile Methoden kommen überwiegend bei einem partizipativen Austausch einer möglichst heterogenen Akteur:innengruppe zur Anwendung; Perspektivenvielfalt und Interdisziplinarität ermöglichen es, komplexe ökologische, wirtschaftliche, soziale und politische Zusammenhänge etwa im Kontext des Klimawandels fassen zu können. Eine solchermaßen wirkende „Gruppenintelligenz" ist sowohl dazu angetan, den Horizont aller Beteiligten zu vergrößern, als auch schwache Signale aus unterschiedlichen Hierarchieebenen einer Organisation oder unterschiedlichen wissenschaftlichen Disziplinen in die Diskussion aufzunehmen. Für einen vertrauensvollen Kommunikations- und Aushandlungsprozess spielt Transparenz im Hinblick auf die Methodik, individuelle Interessen und die allgemeine Zielsetzung eine sehr wichtige Rolle (vgl. Schüll 2015:61; vgl. Dienel 2015:71).

Die Methoden der strategischen Vorausschau, die agil angewendet werden können, sind keinesfalls neue Methoden; sie kommen bereits in unterschiedlichen Kontexten und in verschiedensten Ausprägungen zur Anwendung. Entscheidend ist, dass ihr Forschungsdesign, also die Art und Weise ihrer Umsetzung, an das oben genannte Anforderungsprofil angepasst werden kann. So kann eine Cross-Impact-Analyse systemisch-formal, hochkomplex und langfristig angelegt sein, aber auch anlassbezogen, intuitiv und kurzfristig zu validen Aussagen führen. Agile Methoden eignen sich somit als Ergänzung für langfristig ausgelegte, oft periodisch wiederholte Prozesse der strategischen Vorausschau, um situativ und anlassbezogen Veränderungen wahrzunehmen und auf diese zu reagieren. Zusammenfassend bedeutet dies, dass Methoden der Vorausschau agil anwendbar sind, wenn sie kurzfristig, intuitiv, situativ und partizipativ umsetzbar sind. So können sie die Akteur:innen befähigen, strategische Vorausschau reaktiv, aber auch proaktiv zu betreiben, um schnell zu Orientierung und strategischen Handlungsoptionen zu gelangen.

## *Auswahl agiler Methoden der strategischen Vorausschau*

Die hier vorgestellten Methoden sind im Kontext des Klimawandels besonders dazu geeignet, dem oben genannten Anforderungsprofil zu entsprechen. Anhand der für jede Methode aufgeführten generischen Fragestellungen differenziert sich, welche Methode für welches Erkenntnisinteresse geeignet ist. Damit sie zielführend der Situation entsprechend eingesetzt werden, ist es wichtig, ein Problem abzugrenzen sowie das Erkenntnisinteresse und die Fragestellung zu konkretisieren. Dafür werden sie in die Kategorien „Identifizierung und Bewertung von Umfeldentwicklungen“, „Exploration möglicher Zukunftsbilder und Ableitung von Handlungsoptionen“ und „Definition und Priorisierung von Handlungsoptionen“ unterteilt. Alle die darunter aufgeführten Methoden führen zu strategischen Erkenntnissen.

### *Methoden der Kategorie 1: Identifizierung und Bewertung von Umfeldentwicklungen*

Die Methoden helfen, das Umfeld zu verstehen, auf welche Weise interne und externe Veränderungen zusammenhängen und welche Bedeutung sie für eine:n Akteur:in einnehmen können. Die Methoden aus dieser Gruppe sortieren meist komplex erscheinende Verwirrungen von Entwicklungen. Die Methoden können einzeln eingesetzt werden, um beispielsweise strategische Themen zu ermitteln, sie entfalten jedoch ihre größte Wirkung bei ihrer Anwendung in Methoden der Kategorie 2 und 3.

- Unsicherheit-Wirkungsanalyse (engl. impact-uncertainty analysis) – Welche Bedeutung haben Ereignisse und Entwicklungen im Umfeld der Akteur:innen?

*Ziel:* Die Methode zielt darauf ab, komplexe, diffuse Entwicklungen zu ordnen. Mit dieser Methode können in die Zukunft projizierte Ereignisse wie etwa das Austrocknen wasserführender Schichten in den Wäldern einer Kommune entsprechend ihrer Wirkung und Eintrittswahrscheinlichkeit auf einen Bezugspunkt hin (zum Beispiel Akteur:innen, ein Vorhaben, ein Themenfeld) hinsichtlich ihrer Wirkung und der Wahrscheinlichkeit ihres Eintretens eingeordnet werden. Mit der Methode können Akteur:innen verstehen, auf welche Entwicklungen und Ereignisse sie besonders Augenmerk bei der Ausrichtung ihrer Strategien und Maßnahmen legen müssen (vgl. Pielkahn 2008:431). Die Methode gibt Anlass zur kritischen Reflektion, um sich den Entwicklungen gegenüber (neu) positionieren zu können und ggf. Strategien anzupassen bzw. neue zu entwickeln.[72]

*Anwendung/Bedarf:* Hinsichtlich des Klimawandels und der nachhaltigen Transformation können mit dieser Methode beispielsweise systematisch schwache Signale erkannt werden, die möglicherweise einschneidende Wirkung haben. Aber es können mit ihr auch bekannte Entwicklungen völlig neu bewertet werden. Die Methode kann einzeln oder im Kontext anderer Prozesse der Vorausschau eingesetzt werden, um Informationen aus Recherchen für die Vorausschau (zum Beispiel aus einem Scanning-Prozess) zu sortieren. Diese Methode ermöglicht es, wahrscheinliche Entwicklungen von unwahrscheinlichen zu trennen. Durch die Bewertung ihrer Wirkung werden dabei auch unwahrscheinlichen Entwicklungen im Sinne der Offenheit von Zukunft Beachtung geschenkt. So können „schwache Signale" (Ansoff 1980:132) als Vorboten von sogenannten Wildcards erkannt werden. Wildcards sind Ereignisse mit geringer Wahrscheinlichkeit und sehr hoher Wirkung auf einen Bezugspunkt (vgl. Steinmüller und Steinmüller 2004:18 f.) wie beispielsweise der Ausbruch einer Epidemie oder das Kippen von scheinbar widerstandsfähigen Ökosystemen wie der borealen Wälder. Anschließend wird jede Entwicklung entsprechend ihrer Wirkung auf die Akteur:innen bewertet. Für die Analyse von Wildcards gibt die Literatur zu bedenken, dass sie meist in der beschriebenen Form nicht auftreten, jedoch den Blick für das schärfen, was Akteur:innen nicht wissen können (vgl. Steinmüller 2012:116). Die Methode kann als Kreislauf verstanden werden. Indem sie regelmäßig und situativ eingesetzt

[72] *So kann eine durch Scanning detektierte Entwicklung vor einem Jahr als nicht bedeutsam eingestuft worden sein, muss aber im Hinblick auf aktuelles Geschehen neu bewertet werden.*

wird, kann ein:e Akteur:in einen Überblick über die aktuelle Entwicklung und ihre Bedeutung erlangen.

*Durchführung:* Entsprechend der Fragestellung wird eine Reihe neuer Entwicklungen partizipativ mittels der STEEP-Analyse in den Kategorien Gesellschaft, Technologie, Wirtschaft, Ökologie und Politik (Society, Technology, Economy, Ecology, Policy = STEEP) zusammengetragen (vgl. Steinmüller 2012:132). Die Ergebnisse werden partizipativ in Quadranten eines Koordinatensystems einsortiert, das ein Kontinuum zwischen hoher und niedriger Wirkung und hoher und niedriger Wahrscheinlichkeit ausweist. Die Kategorisierung erfolgt in einem systematisch angelegten Vorgehen und in einer anschließenden Reflexion wird der Handlungsbedarf analysiert und beispielsweise ein Transfer in den Szenarioprozess vorbereitet (vgl. Steinmüller 2012:116). Die Durchführung dauert je nach Umfang der Fragestellung und des Suchfeldes wenige Stunden bis zu zwei Tagen.

*Ergebnis:* Das Ergebnis ist eine Art Landkarte in Form eines Koordinatensystems, das Entwicklungen nach ihrer Bedeutung für die Akteur:innen verortet.

- Wechselwirkungsanalyse (engl. cross-impact analysis) – Wie kann ein Verständnis treibender Kräfte für Entwicklungen im Umfeld gewonnen werden?

*Ziel:* Die Cross-Impact- (oder Wechselwirkungs-)Analyse zielt darauf ab, ein vereinfachtes Systemmodell zu erstellen. Im Kern geht es darum, die Schlüsselfaktoren zu identifizieren, die das zu untersuchende Umfeld, eine:n Akteur:in beeinflussen, und herauszufinden, wie diese Schlüsselfaktoren zusammenhängen. Dabei handelt es sich in der Regel um eine „Grobanalyse" von wechselwirkenden sozialen, politischen, technologischen, ökologischen und ökonomischen Ereignissen/Faktoren. Auf diese Weise liefert die Cross-Impact-Analyse die Grundlage für die Ableitung von plausiblen und vor allem in sich konsistenten Zukunftsentwicklungen in Form von qualitativen Szenarien zur Unterstützung der Strategieentwicklung. Sie basiert auf der Einschätzung von Expert:innen und wird daher vor allem für „Analyseaufgaben eingesetzt, die aufgrund ihrer disziplinären Heterogenität und der Relevanz ‚weichen' Systemwissens keinen Einsatz theoriegestützter Rechenmodelle erlauben, die aber andererseits zu komplex für eine intuitive Systemanalyse sind" (Weimer-Jehle 2014:1). Im Gegensatz zur Unsicherheit-Wirkungsanalyse wird hier die Beziehung von Faktoren zueinander geklärt.

*Anwendung/Bedarf:* Die Cross-Impact-Analyse kommt häufig zur Anwendung, wenn das aktuelle und zukünftige Umfeld einer Organisation analysiert werden soll. Hierbei steht zunächst die Frage im Raum, welche Umfeldfaktoren besonders einflussreich sind bzw. besonders stark durch andere Faktoren beeinflusst werden. Der übergeordnete Anlass für die Cross-Impact-Analyse kann beispielsweise sein, dass mögliche (konsistente) zukünftige Entwicklungen des Organisationsumfelds identifiziert werden sollen. Dieser Möglichkeitsraum kann dann mit den eigenen Planungen und Strategien verglichen werden, um Unsicherheiten oder blinde Flecken aufzuspüren. Zum Beispiel erfordert die Entwicklung regionaler Anpassungsstrategien an den Klimawandel die „Erörterung der Zusammenhänge zwischen Klimatrends und lokalen Vulnerabilitäten“, um auf dieser Grundlage politische Maßnahmen entwickeln zu können (vgl. Weimer-Jehle 2015:243–244).

*Durchführung:* Zu Beginn werden die Systemgrößen oder Faktoren (Deskriptoren) ausgewählt, die betrachtet werden sollen (beispielsweise „Energiepreisentwicklung“ oder „$CO_2$-Preisentwicklung“). In einer Matrix werden anschließend paarweise die gegenseitigen Beeinflussungen zwischen den einzelnen Deskriptoren erhoben, beispielsweise nach dem Schema „verstärkend“, „neutral“ oder „abschwächend“. Die ausgefüllte Cross-Impact-Matrix wird abschließend auf Konsistenz untersucht.

*Ergebnis:* Die ausgefüllte Cross-Impact-Matrix stellt den direkten Output der Methode dar. Sie bildet ein vereinfachtes Systemmodell ab, auf das sich die beteiligten Expert:innen einigen konnten und enthält zahlreiche Ansatzpunkte zur Information von Strategieprozessen. Zum Beispiel können aus den konsistenten Matrixausprägungen alternative qualitative Szenarien für das Organisationsumfeld abgeleitet werden.

Die beiden Methoden der Kategorie 1 unterscheiden sich darin, dass die Wechselwirkungsanalyse Zusammenhänge zwischen Faktoren und Entwicklungen erklärt, während über die Unsicherheit-Wirkungsanalyse jede Entwicklung für sich auf Eintrittswahrscheinlichkeit sowie die Stärke der Wirkung auf die Akteur:innen oder deren Umfeld eingeschätzt werden kann (s. Abb. 11.2).

*Methode der Kategorie 2: Exploration möglicher Zukunftsbilder und Ableitung von Handlungsoptionen*

Die Methode dieser Kategorie verknüpft Entwicklungen miteinander logisch und plausibel, um mögliche zukünftige Zustände und deren Alternativen aus belegbaren Fakten zu konstruieren. Sie dient dazu, Handlungsräume aufzuzeigen und Handlungsoptionen abzuleiten.

- Explorative Szenarioanalyse – Auf welche Veränderungen kann sich ein:e Akteur:in einstellen?

*Ziel:* In dieser Form der Szenarioanalyse werden explorative Szenarien erarbeitet. „Szenarien der Zukunftsforschung sind auf wissenschaftliche Weise generierte Zukunftsbilder“ (Steinmüller 2012:108 f.), die auf Fakten basieren, jedoch nicht faktisch oder prognostisch sind. Explorative Szenarien dienen dazu, ein breites Spektrum von möglichen, nicht aber wahrscheinlichen Zukünften diskutieren zu

| **Ziel: Identifizierung und Bewertung von Umfeldentwicklungen** | | |
|---|---|---|
| **Methode** | **Beispielfragestellung** | **Ergebnis** |
| Wechselwirkungsanalyse  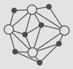 | Wie beeinflussen Digitalisierung und die lokalen Auswirkungen des Klimawandels die $CO_2$-Emissionen einer Kommune? | • Kenntnis direkter/indirekter Zusammenhänge über ein Systemmodel<br>• Anknüpfungspunkte für strategische Handlungsoptionen |
| Unsicherheit-Wirkungsanalyse  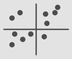 | Mit welcher Wahrscheinlichkeit und Stärke wirken mögliche Ereignisse, wie Hochwasser, und Entwicklungen, wie langanhaltende Hitze, auf einen Produktionsbetrieb? | Kategorisierung von Entwicklungen und Ereignisse in einer Matrix entsprechend ihrer Eintrittswahrscheinlichkeit und Wirkung |

*Abb. 11.2 Gegenüberstellung der Wechselwirkungsanalyse und Unsicherheit-Wirkungsanalyse anhand von Beispielfragen sowie ihrer Ergebnisse*

können. Szenarioanalysen sind zum Teil sehr umfangreiche, systemisch-formale Prozesse. Die agile explorative Szenarioanalyse zielt auf die Entwicklung von Szenarien in partizipativer Form in kurzer Zeit ab, um anhand dieser Handlungsoptionen aufzuzeigen und Strategien ableiten zu können. Damit ist es möglich, sich auf eine Vielzahl möglicher Entwicklungen vorzubereiten (Steinmüller 2012:110 ff.).

*Anwendung/Bedarf:* Die agile explorative Szenarioanalyse wird angewendet, um kurzfristige Entwicklungen und deren Auswirkung auf die Zukunft zu prospektieren und damit Handlungsorientierung zu erlangen. Gerade aufgrund der dynamischen Veränderungen im Zuge des Klimawandels ist es wichtig, dass Akteur:innen unterschiedlicher Ebenen einer Organisation deren Auswirkungen im Kontext ihrer spezifischen Fragestellungen bewerten können. Mittels der Methode lassen sich drei verschiedene Fokusse setzen (s. Abb. 11.3): Über Umfeldszenarien können Akteur:innen nachvollziehen, wie sich die Welt außerhalb wandelt: Aus Sicht eines Unternehmens zum Beispiel die Veränderungen in naheliegenden Branchen, die direkt von Hitzewellen und Ernteausfällen betroffen sind oder aus Sicht einer Kommune die Veränderungen im Alltag der Bürger:innen einer Stadt aufgrund von Klimaveränderungen. Durch Kontextszenarien wird ein Verständnis dafür gewonnen, welchen Einfluss Umfeldentwicklungen auf die Akteur:innen haben können (zum Beispiel klimabedingte Rohstoffverknappung auf die Zulieferung entlang der Wertschöpfungskette und Herstellung). Eine gesonderte Form der Szenarien sind strategische Szenarien, die die Auswirkungen von bestimmten

Strategien einer Akteurin oder eines Akteurs auf das Umfeld testen (beispielsweise ob neue Produktideen deren nachhaltige Nutzung bei Kund:innen motivieren oder es zu ungewollten Nebenfolgen kommt). Die Szenarioanalyse ermöglicht es, ein Spektrum von Handlungsspielräumen aufzuzeigen, (zum Beispiel durch Neubewertung von äußeren Gegebenheiten), auf diese agil reagieren, aber auch proaktiv gestalten zu können und somit Entwicklungen in einem bestimmten Rahmen entsprechend eigener Vorstellung zu beeinflussen.

*Durchführung:* Mittels der agilen Szenarioanalyse werden Verknüpfungen von wissenschaftlich fundierten Projektionen zu Schlüsselfaktoren innerhalb eines abzugrenzenden Themenfeldes gebildet. Dazu werden die Verknüpfungen narrativ plausibilisiert und zu komplexen Szenarien angereichert.[73] Diese müssen in sich konsistent sein, sich jedoch voneinander stark unterscheiden. Die Szenarioanalyse greift dazu andere bewährte Methoden auf, zum Beispiel die Cross-Impact-Analyse. In einer Reflexion werden Handlungsspielräume identifiziert und dann hinsichtlich ihrer Relevanz und Wünschbarkeit bewertet, um die Erkenntnisse in den Strategieprozess geben zu können (vgl. Steinmüller 2012:110 f.)[74]. Ein:e Moderator:in hilft den Teilnehmenden die Schritte durchzuführen und sie zu visualisieren. Der Prozess kann innerhalb weniger Wochen, aber auch innerhalb von zwei bis drei Tagen durchgeführt werden.

*Ergebnis:* Meist werden drei bis fünf Szenarien entwickelt. Ein weiteres wichtiges Ergebnis ist die Bewertung der Szenarien in Bezug auf Handlungsoptionen. Auf diese kann später zurückgegriffen werden, falls sich Entwicklungen ergeben, die einem bestimmten Szenario ähneln. Das dritte Ergebnis sind Strategien, mit denen sich ein:e Akteur:in gegenüber bestimmten präferierten oder im besten Fall gar allen Szenarien aufstellen kann.

*Methoden der Kategorie 3: Definition und Priorisierung von Handlungsoptionen*

Die Methoden dieser Kategorie tragen dazu bei, Visionen und Zielsetzungen entsprechend äußerer Entwicklungen und Rahmenbedingungen zu erarbeiten, anhand derer konkrete Strategien erarbeitet werden können und somit die Zukunft gestaltbar wird.

[73] *Hochformalisierte Szenarioprozesse hingegen berechnen die Konsistenz zwischen den Projektionen, was je nach Anzahl von Faktoren längere Zeit in Anspruch nehmen kann.*

[74] *Dieser achtstufige Prozess findet sich verschiedenfach in der Literatur und wurde von Mietzner und Reger (2005:230 ff.) verglichen.*

| Ziel: Exploration möglicher Zukunftsbilder und Ableitung von Handlungsoptionen | | |
|---|---|---|
| **Methode** | **Beispielfragestellung** | **Ergebnis** |
| Explorative Szenarioanalyse  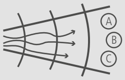 | • Welche alternativen Konsumentwicklungen sind im Umfeld einer Branche möglich?<br>• Auf welche möglichen Zukunftsszenarien kann sich eine Kommune im Kontext von Entwicklungen in der Wasserversorgung vorbereiten?<br>• Welchen Einfluss kann die Strategie der situationsbezogenen Fahrverbote auf die Klimabilanz und Mobilitätsverhalten der Menschen ausüben? | Kenntnis von Handlungsoptionen mittels eines Spektrums unterschiedlicher Szenarien |

*Abb. 11.3 Differenzierung des Fokus der explorativen Szenarioanalyse anhand von Beispielfragestellungen auf Kontext, Umfeld oder Strategie der Akteur:innen*

- Visual Roadmapping – Das Ziel steht fest, wie ist der Weg dahin?

*Ziel:* Die Visual-Roadmapping-Methode ist eine vom Institut für Innovation und Technik (iit) in der VDI/VDE Innovation + Technik GmbH entwickelte Methode, die sich besonders für die Vorausschau und Identifizierung von verschiedenen Meilensteinen oder Schlüsselmaßnahmen auf dem Weg vom „Jetztzustand" hin zu einem normativen Ziel eignet, das mittel- bis langfristig in der Zukunft liegt (vgl. Bovenschulte et al. 2011:1). Anhand der Methode können mögliche strategische Handlungspfade visualisiert und Wechselwirkungen zwischen Schlüsselmaßnahmen und Umfeldfaktoren identifiziert werden. Ziel ist also, einen Blick in eine gewünschte Zukunft zu werfen sowie gleichzeitig von einem Zielpunkt in der Zukunft im Sinne eines „Backcastings" zurückzudenken, um mögliche Handlungspfade zu identifizieren.

*Anwendung/Bedarf:* Die Visual-Roadmapping-Methode wird in der Regel eingesetzt, wenn Klarheit über ein mittel- bis langfristiges politisches oder unternehmerisches Ziel besteht, der Umsetzungsweg bzw. strategische Schlüsselmaßnahmen jedoch noch identifiziert oder priorisiert werden müssen. Zum Beispiel kann ein solches Ziel die Klimaneutralität einer Kommune oder die Einführung einer nachhaltigen Produktlinie sein. Entsprechend kann eine weitere Anwendung sein, mögliche angedachte strategische Maßnahmen sowie implizite Annahmen über zukünftige Umfeldentwicklungen im Diskurs zu überprüfen und ggf. zu validieren sowie alternative Handlungsstränge aufzuzeigen.

*Durchführung:* Im Diskurs werden jene Schlüsselmaßnahmen oder möglichen Handlungspfade identifiziert, die nach Einschätzung der Expert:innen wichtige Meilensteine zur Erreichung des übergeordneten Ziels darstellen. Diese werden auf einer Zeitachse verordnet und vier Kategorien zugeordnet: (1) sozioökonomische und regulative Rahmenbedingungen, (2) wissenschaftliche und technische Entwicklungen, (3) Entwicklung von Produkten und Dienstleistungen sowie (4) wirtschaftliche und gesellschaftliche Auswirkungen. Durch die unmittelbare Visualisierung der Schlüsselmaßnahmen und weiterer zentraler Umfeldfaktoren können zudem signifikante Wechselwirkungen (Synergien, Hemmungen) zwischen diesen erfasst und etwaige Inkonsistenzen ausgeräumt werden.

*Ergebnis:* Bei der Visual Roadmap handelt es sich um einen Strukturplan, der potenzielle Handlungspfade und deren Zusammenhänge/Abhängigkeiten eingeordnet in einen zeitlichen Verlauf leicht handhabbar aufzeigt, sodass Handlungsempfehlungen daraus abgeleitet werden können (vgl. Bovenschulte et al. 2011:3).

- Agiler Roadmapping-Prozess – Was ist das Ziel und wie ist der Weg dahin?

*Ziel:* In einem Roadmapping-Prozess einigen sich verschiedene Akteur:innen auf Ziele und Aktivitäten wie zum Beispiel die Bearbeitung von externen und internen Herausforderungen innerhalb eines zuvor abgegrenzten Themenbereichs und Zeithorizonts. Die Roadmap dient dazu, eine gemeinsame Vision, ein Verständnis der aktuellen Situation (intern und extern) sowie von Barrieren auf dem Weg zur Vision zu gewinnen. Auf diese Weise können Strategien zum Erreichen der Vision/der Ziele abgeleitet und Handlungen koordiniert werden. Roadmaps sind kein statisches Planungswerkzeug, sondern können in agiler Form angepasst werden, wenn sich Bedingungen ändern. Durch geeignete Visualisierung können die Teilnehmenden jederzeit auf Ergebnisse zurückgreifen (vgl. Pielkahn 2008:321 f.). Der agile Roadmapping-Prozess sieht den Ergebnisgewinn in kurzer Zeit vor ohne nachgelagerte Validierung sowie deren Moderation in klassischen Prozessen.

*Anwendung/Bedarf:* Die Anwendungsmöglichkeiten von agilen Roadmapping-Prozessen sind vielfältig. So können Visionen mit Akteur:innen erarbeitet werden, die vorher noch nie zusammengearbeitet haben, aber aufgrund des Klimawandels nun zusammenarbeiten müssen (zum Beispiel verschiedene Verkehrsträger). Mittels des Roadmapping-Prozesses lassen sich Strategien entwickeln, beispielsweise wann und welche Technologien im Zuge äußerer Einflüsse, wie steigende Temperaturen, erforscht und entwickelt werden müssen oder wann und mit welchen Maßnahmen unterschiedliche Kräfte in koordinierter Weise zu bestimmten Zeitpunkten Synergien hinsichtlich der Ziele erzeugen (vgl. Pielkahn 2008:321 f.).

| Ziel: Definition und Priorisierung von Handlungsoptionen | | |
|---|---|---|
| **Methode** | **Beispielfragestellung** | **Ergebnis** |
| Visual Roadmapping  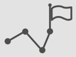 | Durch welche Schlüsselmaßnahmen kann eine Kommune ihr Ziel der Halbierung lokaler $CO_2$-Emissionen erreichen und wie können diese Maßnahmen priorisiert werden? | Definierte und priorisierte Schlüsselmaßnahmen entsprechend des Ziels |
| Agiler Roadmapping-Prozess  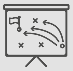 | Welche gemeinsamen Ziele und Strategien können Kommunen, lokale Wirtschaft und Zivilgesellschaft entwickeln, um ihre Region nachhaltiger zu gestalten? | Abgestimmte Visionen, Ziele, Aktivitäten und Verantwortlichkeiten in einer Roadmap |

*Abb. 11.4 Differenzierung des Visual Roadmapping und des agilen Roadmapping-Prozesses anhand von Beispielfragestellungen und ihrer Ergebnisse*

*Durchführung:* Für den Roadmapping-Prozess ist es empfehlenswert, eine:n Moderator:in zu bestimmen, welche:r die Teilnehmenden durch den Prozess führt und Ergebnisse visuell dokumentiert. Die Teilnehmenden einigen sich auf eine Vision und auf Ziele. Nach einer Status-Quo-Analyse werden Barrieren auf dem Weg zu den Zielen identifiziert, um sodann mit den internen und externen Gegebenheiten Meilensteine und Maßnahmen abzuleiten, zeitlich zu priorisieren und die Beteiligten in ihrer jeweiligen Rolle zuzuordnen. Die Durchführung des Prozesses dauert zwei bis drei Tage.

*Ergebnis:* Je nach Schwerpunkt der Fragestellung handelt es sich um eine strategische visualisierte Roadmap, die Aktivitäten unterschiedlicher Akteur:innen auf einer Zeitleiste verortet, oder eine Forschungs- und Entwicklungsroadmap, die Forschungsthemen priorisiert.

Wie in Abb. 11.4 veranschaulicht, besteht der Unterschied der beiden Methoden dieser Kategorie neben den Schritten der Durchführung darin, dass beim Visual Roadmapping bereits ein Ziel (zum Beispiel per Gesetz) vorgegeben ist, für das Handlungspfade gesucht werden. Beim agilen Roadmapping-Prozess kann das Ziel für ein künftiges Vorhaben durch die Teilnehmenden auch gemeinsam bestimmt werden, ehe Handlungspfade erarbeitet werden.

Agile Methoden der strategischen Vorausschau können einzeln angewendet werden, um daraus spezifische strategische Ergebnisse abzuleiten. Sie können sich aber auch in Kombination sinnvoll ergänzen. Während sich einige dazu eignen, Entwicklungen

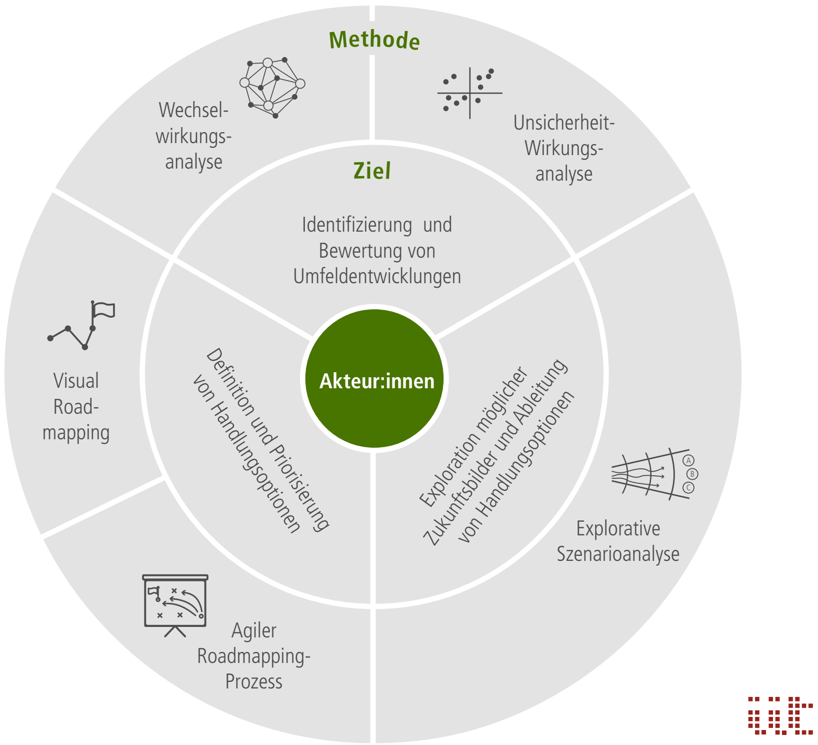

*Abb. 11.5 Gegenüberstellung von möglichen Zielstellungen von Akteur:innen sowie Zuordnung entsprechender Methoden*

im Umfeld zu bewerten, können andere darauf aufbauen, um Handlungsoptionen aufzuzeigen und zu priorisieren. (s. Abb. 11.5).

## *Handlungsbedarf und Ausblick*

Der Klimawandel wird weltweit zu massiven gesellschaftlichen und wirtschaftlichen Umbrüchen führen. Dabei ist davon auszugehen, dass bislang nur ein kleiner Teil der möglichen Auswirkungen abschätzbar ist. Vor allem komplexe Kaskadeneffekte, etwa die Freisetzung großer Mengen an zusätzlichen Treibhausgasen durch auftauende Permafrostböden, sind bislang kaum abzusehen (vgl. Lenton et al. 2019). Massive direkte Auswirkungen des Klimawandels wie Stürme, Hitze- und Dürrewellen oder Wassermangel sind auch in Deutschland höchst wahrscheinlich (vgl. Hoegh-Guldberg et al. 2018) – und das nicht erst in 70 Jahren, sondern bereits in diesem Jahrzehnt (s. Kap. 3: Klimakonsequenzen für die Natur und den Menschen). Indirekte wirtschaftliche und gesellschaftliche Auswirkungen sind bereits jetzt zu spüren. Selbst wenn es die Weltgemeinschaft schaffen sollte, in den nächsten 30 Jahren global für eine Netto-Null-Emission zu sorgen, wie vom Weltklimarat IPCC gefordert, werden sich eine Vielzahl an Klimafolgen nicht vermeiden lassen (Lenton et al. 2019). Die resultierenden Folgen werden etablierte Lebens- und Arbeitsweisen – und entsprechende strategische Planungen – fortlaufend radikal in Frage stellen. Die hier aufgezeigten Methoden können dazu genutzt werden, das Dilemma zwischen Handlungsdruck und fehlender Handlungsorientierung in Zeiten großer Unsicherheit zu mildern. Kommunen sowie kleine und mittlere Unternehmen stehen unter besonders hohem Handlungsdruck, ihre Strategieprozesse durch situativ einsetzbare Methoden zu ergänzen.

Da die Zukunft prinzipiell offen ist, können Resultate der strategischen Vorausschau natürlich nicht die Zukunft abbilden, aber sie können Orientierungswissen bieten. Um agile Methoden der strategischen Vorausschau einzusetzen und den aufkommenden Veränderungen proaktiv zu begegnen, bedarf es einer vorausschauenden Denkweise, die die Offenheit der Zukunft akzeptiert und nutzt. Mit dieser offenen Denkweise kann in Alternativen gedacht werden. Auf diese Weise können Handlungsspielräume in partizipatorischen Prozessen diskutiert, aufgezeigt und dann mit konkreten Maßnahmen umgesetzt werden. Wie bereits in einigen Organisationen institutionalisiert, können diese Denkweise und agile Methoden Akteur:innen auf allen Ebenen der Organisation dabei unterstützen, zukunftsgerichtete Fragestellungen situativ und flexibel zu beantworten. Dabei wirken agile Methoden der strategischen Vorausschau ergänzend und ersetzen keine formalisierten Vorausschauprozesse wie das Monitoring und Scanning der Umwelt.

Um mit den oben genannten Umbrüchen zurechtzukommen und Lösungen zum Erreichen der Netto-Null zu erarbeiten, ist agiles strategisches Handeln gefragt. Das Konzept, Prozesse der Vorausschau dabei so anzulegen, dass sie kurzfristig zu Ergebnissen führen, ist keinesfalls neu. Vor dem Hintergrund des Klimawandels und der Diskussion um die Ausweitung der Debatte über agiles Management hinsichtlich einer durch Reflexion geförderten Entscheidungsfähigkeit erlangen agile Methoden der Vorausschau jedoch eine neue Bedeutung. Sie ermöglichen allen Akteur:innen, die Fähigkeit der wissenschaftlich fundierten Vorausschau zu erlernen, um auf allen Ebenen einer Organisation entscheidungsfähig zu sein. Damit wird es möglich, die Anpassung an Klimawandel und Klimapolitik – im Sinne einer nachhaltigen und resilienten Transformation – nicht nur als Zwang, sondern als Handlungsspielraum für proaktive Zukunftsgestaltung aufzufassen.
